# Origin of the retroviruses: when, where, and how?

**DOI:** 10.1016/j.coviro.2017.06.006

**Published:** 2017-08

**Authors:** Alexander Hayward

**Affiliations:** Centre for Ecology and Conservation, University of Exeter, Cornwall Campus, Penryn, TR10 9FE, United Kingdom

## Abstract

Retroviruses are a virus family of considerable medical and veterinary importance. Additionally, it is now clear that endogenous retroviruses (ERVs) comprise significant portions of vertebrate genomes. Until recently, very little was known about the deep evolutionary origins of retroviruses. However, advances in genomics and bioinformatics have opened the way for great strides in understanding. Recent research employing a wide variety of bioinformatic approaches has demonstrated that retroviruses evolved during the early Palaeozoic Era, between 460 and 550 million years ago, providing the oldest inferred date estimate for any virus group. This finding presents an important framework to investigate the evolutionary transitions that led to the emergence of the retroviruses, offering potential insights into the infectious origins of a major group of pathogenic viruses.

## Highlights

• Retroviruses are a virus family of considerable medical and veterinary importance.
• Until recently, very little was known about deep retroviral origins.
• New research supports a marine origin of retroviruses, ∼460–550 million years ago.
• The evolutionary events leading to the origin of retroviruses remain obscure.
• Improved understanding of Metaviridae diversity and evolution are required for this.

**Current Opinion in Virology** 2017, **25**:23–27

This review comes from a themed issue on **Paleovirology**

Edited by **Aris Katzourakis**

For a complete overview see the Issue and the Editorial

Available online 30th June 2017

**http://dx.doi.org/10.1016/j.coviro.2017.06.006**

1879-6257/© 2017 The Author. Published by Elsevier B.V. This is an open access article under the CC BY license (http://creativecommons.org/licenses/by/4.0/).

## Introduction

The origin of species was famously described as ‘that mystery of mysteries’ by the 19th Century scientist Sir Charles Lyell. Since Charles Darwin presented his theory of natural selection, thereby solving the mystery, much progress has been made in understanding speciation. But a major challenge remains elucidating the evolutionary transitions leading to the emergence of whole new groups of taxa. This is especially so for viruses, with their vast diversity and multiple independent origins. Unravelling viral beginnings is particularly challenging because viruses typically have small genomes, rapid rates of sequence evolution, and scant fossil remains. Yet, over recent decades it has become apparent that viruses occasionally leave a historical record in their host's genomes in the form of endogenous viral elements (EVEs) [1, 2], which can provide valuable information for discerning their histories. As a consequence of their lifecycle, this record is particularly rich for retroviruses. To replicate, a retrovirus must integrate a copy of its genome into the host's genome as a provirus. If this occurs in a host germ cell, the provirus may be inherited by the host's progeny and down the generations as an endogenous retrovirus (ERV). All vertebrate genomes examined so far contain ERVs, providing a wealth of opportunities to study retroviral ecology and evolution, including the origin of this medically and veterinarily important viral group [3^•^]. In combination with modern genomic technologies, analysis of ERV sequences is facilitating rapid advances in the understanding of retroviral evolution. Here, we review recent progress in discerning retroviral origins, outlining key advances and highlighting outstanding research questions (Box 1).

Box 1 **Retroviruses**

Retroviruses form a family of enveloped RNA viruses (the Retroviridae), entirely restricted to vertebrate hosts [4]. Exogenous retroviruses (XRVs) are transmitted horizontally among hosts. The number of described XRVs is small, and is distributed across 7 genera, containing just 53 described species [5]. Meanwhile, ERVs, which are inherited vertically in the genomes of their hosts, represent a great wealth of retroviral sequence diversity that has accumulated over millions of years of vertebrate–retrovirus interactions. ERVs accumulate mutations at the background rate of sequence mutation in their host's genome, gradually degrading until their sequences are no longer recognisable. ERV taxonomy is not covered by the guidelines of the International Committee on Taxonomy of Viruses (ICTV). Consequently, here we refer to ERVs as retrovirus‘-like’, with reference to their placement in phylogenetic analyses [3•, 6].

## The when, where, and how of retrovirus origins

### When did retroviruses originate?

Several methods have been employed to estimate the age of retroviral lineages over evolutionary timescales. These approaches can be divided broadly into those that reconstruct ERV integration histories, and those that analyse host–virus co-phylogenetic relationships. We discuss the relevance of these for examining ancient retroviral origins below, providing a brief overview of recent developments of particular significance.

When a retrovirus undergoes reverse transcription to produce viral DNA, identical sequences called long terminal repeats (LTRs) are generated at both ends of the viral genome [4]. After integration, LTRs diverge independently at a rate approximately equal to the background (neutral) rate of host genome sequence evolution [7]. Consequently, measures based on divergence between 5′ and 3′ LTRs can be used to calculate approximate proviral integration dates by adopting the formula *t* = *k*/2*N*, where *t* is time, *k* is LTR divergence in substitutions per site, and *N* is the neutral rate of evolution for the host genome. This approach has been widely applied to estimate integration dates for a range of ERV lineages, from diverse host genomes, including primates [8, 9], bats [10], amphibians [11, 12••] and fish [12^••^]. However, gene conversion and recombination between LTRs can mislead results [13]. Further, while LTR divergence estimates can be accurate over relatively recent timescales, poor data for neutral mutation rates in ancestral host genomes and the cumulative effects of sequence erosion over ancient time periods limit the utility of this method for addressing the ultimate origins of retroviruses.

An alternative approach to estimate the age of retroviral lineages is to screen host genomes for orthologous ERVs (those shared due to integration in a common ancestor). Since multiple integrations and fixations of ERVs at the exact same location are extremely unlikely, a shared ERV locus is strong evidence of inheritance from the common ancestor where the original germ line integration occurred. Thus, identifying the node in host phylogeny whereby descendent taxa share an orthologous ERV can provide a minimum integration estimate. Using this approach, analysis of lenti-like ERVs orthologous to RELIK (the first identified endogenous lentiretrovirus [14]) from across the order Lagomorpha, provided a minimum age of ∼12 million years for integration of the ancestral element [15]. Similarly, analyses of ERV-L elements orthologous across placental mammals placed integration of an ancestral element at 104–110 mya [16^••^]. However, ‘look-back time’ using orthologues is limited by computational and practical difficulties associated with recovering ERV and host flanking sequences [16^••^], and no older orthologous ERVs have yet been identified. A similar approach, employing host genome duplication events instead of orthology, can also be used to date retroviral lineages [17^•^], but is subject to the same limitations.

The age of ancient retroviral lineages can also be estimated by considering host–virus coevolutionary relationships. Since host switching is frequent among many retroviral lineages [3•, 18], such analyses are largely restricted to spumaretroviruses (foamy viruses), which show relatively few cross-species transmission events [19••, 20]. Additionally, spumaretroviruses are the most basal currently recognised retroviral genus [3^•^], providing a suitable group with which to explore retroviral origins. Incorporation of ancient spuma-like ERVs from sloth genomes (SloEFVs) and exogenous spumaretroviruses isolated from diverse mammalian hosts in several analyses have supported strict host–virus cophylogeny, establishing that spumaviruses are at least 100 million years old [17^•^]. More recently, a study that included ERVs screened from a wide diversity of host taxa from primitive vertebrate lineages, provided an estimated date of origin for spumaretroviruses/spuma-like viruses of ∼455–473 mya during the Ordovician [12^••^]. This suggests that retroviruses emerged alongside their vertebrate hosts during the early Palaeozoic Era, ∼460–550 mya [12^••^]. Such an ancient postulated origin constitutes an exciting development in the study of retrovirology, and virology more generally, providing the oldest date directly inferred for any viral group [12^••^].

### Where did retroviruses originate?

A retroviral origin during the Ordovician period or earlier means that retroviruses must have evolved within the marine environment [12^••^]. Vertebrates were wholly restricted to the sea during the Ordovician, and the first tetrapods did not evolve until the late Devonian. Even considering the possibility that retroviruses evolved outside of the Vertebrata, life on land during the Ordovician was extremely limited, making it unlikely that retroviral origins lie anywhere other than the World's ancient oceans.

It is currently unclear in which host lineage retroviruses originated. The more basal branches of retrovirus phylogeny remain poorly resolved [3•, 12••]. However, given broad congruence between phylogeny for spumaretroviruses and their hosts, and date estimates calculated using rates of viral evolution [12^••^], it is probable that retroviruses were already present before the most recent common ancestor of the chondrichthyans (sharks, rays and chimaeras) and osteichthyans (bony fishes and tetrapods). A small number of ERVs that form a lineage lying more basal to the spumaretroviruses are present in the genome of the sea lamprey, which belongs to a jawless fish lineage estimated to have evolved ∼500 mya [3^•^]. However, these ERVs may originate from a host-switching event rather than being modern descendants of early retroviruses [3^•^].

### How did retroviruses originate?

Retroviruses bear much similarity to transposable elements in the LTR retrotransposon group. For example, both groups share a similar genomic content, including LTRs and group specific antigen (*gag*) and polymerase (*pol*) genes. Indeed, the family Retroviridae is generally classified within the LTR retrotransposon group, alongside Metaviridae (Ty3-gypsy-like elements), Pseudoviridae (Ty1-copia-like elements), and Bel-Pao elements [5, 21, 22]. Retroviral sequences share most similarity with Ty3-gypsy-like elements, and early phylogenetic analyses suggested that Metaviridae is the group most closely related to Retroviridae [23•, 24, 25•], which remains the prevailing view [5, 26]. A commonly stated difference between Retroviridae and Metaviridae is that retroviral elements possess an envelope gene (*env*, coding for transmembrane and host receptor binding proteins that facilitate viral transmission and infection [4]). However, this distinction is somewhat blurry, since ERVs often loose *env* [27], essentially making them analogous to LTR-retrotransposons. Meanwhile, multiple evolutionary transitions are apparent in several Ty3-gypsy-like lineages, whereby a third open reading frame coding for an *env*-like gene allows Ty3-gypsy-like elements to act similarly to infectious retroviruses [28, 29]. The retroviral *env* gene appears to have a different origin to *env*-like genes examined from the Metaviridae, which are proposed to have diverse origins [28, 29]. Specifically, it is hypothesised that there have been at least three acquisitions of an envelope-like gene from disparate viral sources in Metaviridae, with: insect errantiviruses (i.e. gypsy and closely related lineages) acquiring an envelope-like gene from a class of insect baculoviruses (double-stranded DNA viruses with no RNA intermediate); Cer elements acquiring an envelope-like gene from phleboviruses (a genus of negative-sense single-stranded RNA viruses in the family Bunyaviridae); and Tas elements potentially acquiring an envelope-like gene from herpesviruses (double-stranded DNA viruses with no RNA intermediate). The realisation that some lineages in Metaviridae include members with envelope-like genes has led to some murkiness in nomenclature, with Ty3-gypsy-like elements containing *env*-like genes sometimes being referred to as retroviruses [29, 30, 31, 32, 33, 34]. Meanwhile, the pararetroviruses, although closely related to LTR retrotransposons [35], are reverse transcribing viruses that possess a viral genome consisting of a circular double stranded DNA molecule, that do not require integration into the host genome for replication. The pararetroviruses include the enveloped Hepadnaviridae, which infect vertebrates (e.g. Hepatitis B virus), and the Caulimoviridae, which infect plants (e.g. Cauliflower mosaic virus, CaMV).

**Figure 1:**
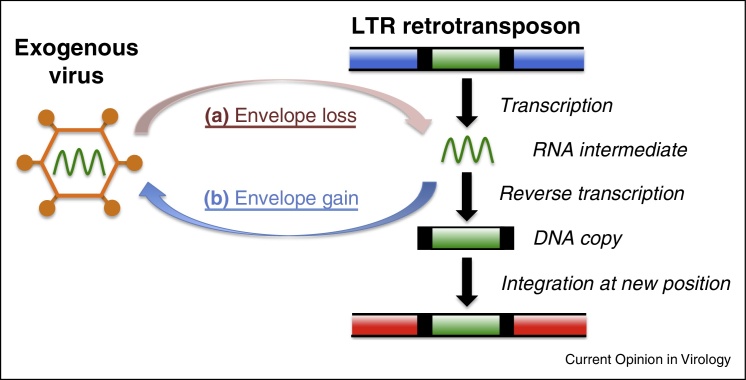
Which came first, the virus or the retrotransposon? LTR transposons may have evolved from a viral ancestor by loosing an envelope gene (with subsequent gains of env-like genes in some cases) **(a)**. Alternatively, Retroviruses may have evolved from an LTR transposon ancestor by gaining an envelope gene **(b)**.

**Figure 2:**
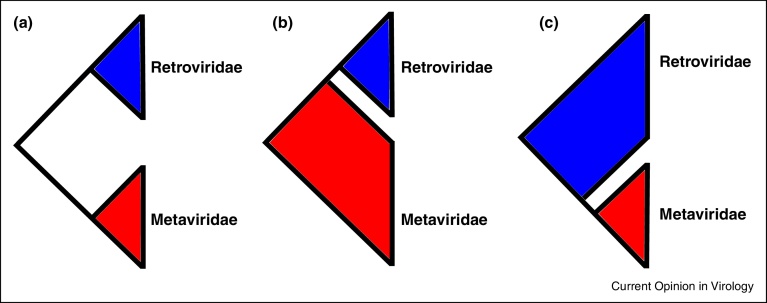
Three key hypotheses to elucidate the ultimate evolutionary origins of Retroviridae: **(a)** Retroviridae is sister to Metaviridae, **(b)** Retroviridae originates from a paraphyletic Metaviridae, or **(c)** Metaviridae originates from a paraphyletic Retroviridae.

The close relationship between Metaviridae and Retroviridae presents a kind of evolutionary chicken-and-egg scenario over which came first, since Ty3-gypsy-like elements may have evolved from an exogenous viral ancestor and lost their *env* gene (with subsequent independent gains of *env*-like genes in certain lineages), or retroviruses may have evolved from an LTR retrotransposon ancestor and gained an *env* gene (Figure 1). Given the extremely deep timescales involved, so far it has not been possible to explicitly resolve the question using outgroup comparison to root the Metaviridae-Retroviridae association (e.g. using members of the Pseudoviridae). Thus, considering the ultimate origins of the retroviruses, the above framework presents three alternative hypotheses: (i) Retroviridae forms a sister group to the Metaviridae, (ii) Retroviridae originates from a lineage within the Metaviridae (i.e. Metaviridae is paraphyletic), or, (iii) Metaviridae originates from a lineage within the Retroviridae (i.e. Retroviridae is paraphyletic) (Figure 2). Retroviridae is entirely restricted to vertebrate hosts, whereas Metaviridae has a wide range of hosts from across the Eukaryota [5]. Consequently, it is tempting to speculate that Retroviridae originates from a lineage within the Metaviridae, that flourished alongside the evolution of its vertebrate hosts in early Palaeozoic seas. However, the situation could be yet more complicated. For example, some authors have suggested that Retroviridae is polyphyletic, comprising of at least three separate lineages that each share a closer relationship with a lineage of Ty3-gypsy-like element [36].

A key limitation to resolving the origin of retroviruses is that testing among competing hypotheses requires a deep-time phylogenetic approach, at the very limit of existing methods of analysis. Current approaches to estimating retroviral phylogeny typically employ alignments based on a contiguous section of the pol gene. While these methods work well for estimating retroviral phylogeny over relatively recent timescales and among closely related taxa, the rapid rate of retrovirus sequence evolution generally means that they do not perform well over deep evolutionary timescales or diverse sets of taxa. Recently, an alternative approach utilising multiple short motifs from across the retroviral genome was developed (with the exclusion of *env*, which evolves too rapidly to be useful at deep timescales), and utilised to infer broad-scale evolutionary patterns across retroviral diversity [3•, 18, 37]. By focussing on short, slowly evolving sequences associated with essential retroviral functions, such as the active sites of core enzymes, this approach offers a means of reducing noise and maximising signal in phylogenetic datasets. It is likely that similar approaches may offer a useful means of disentangling the evolutionary relationships leading to the origin of the Retroviridae in the future. Such approaches necessarily avoid the utilisation of *env* because: (i) the surface component (SU) of the retroviral envelope is the interface between host and virus, and is subject to particularly high substitution rates as a consequence of ongoing host–virus antagonistic coevolutionary cycles; and, (ii) phylogenetic analyses have provided evidence of the promiscuous acquisition of *env*, whereby recombination events lead to the envelope gene having a separate evolutionary history from the rest of the retroviral genome (i.e. so-called ‘*env*-swapping’ or ‘*env*-snatching’) [38, 39, 40].

## Conclusions and future directions

Recent years have seen great developments in our understanding of retrovirus biology, including a deeper knowledge of retroviral phylogeny, diversity, and evolution. This has been fuelled by rapid advancements in genomics and bioinformatics, driving novel insights into what is traditionally considered to be a difficult group. High among the list of achievements is a much improved understanding of the evolution and origin of retroviral lineages, facilitated, for example, by rigorous screening of diverse host genomes and the development of novel and elegant forms of analysis [12^••^]. However, many challenges remain to more fully elucidate the deep evolutionary origins of retroviruses. More basal regions of retrovirus phylogeny are still relatively poorly known, with whole new retroviral clades being discovered and requiring better characterisation [3•, 12••]. Improving knowledge of phylogenetic relationships in this region of retroviral phylogeny may help to narrow down the ultimate date of retroviral origin, and illuminate the host lineages in which the first retroviruses evolved. Additionally, greater scrutiny of the evolutionary transitions leading up to the origin of the retroviruses is required. A current poor understanding of Metaviridae phylogeny complicates this. Further work to reconstruct the phylogeny of Metaviridae is also important to trace the evolution of the retroviral envelope gene and env-like genes in Ty3-gypsy-like elements, which is an enigmatic topic of potential medical importance. Further progress in these areas will likely require genomic screening of even greater swathes of host taxonomic diversity, alongside continued methodological developments. But as is clear from recent progress, persistence offers considerable scope for significant advancements, making the elucidation of retroviral origins an exciting research field to work in.

## Conflict of interest statement

The authors declare no conflict of interest.

## References and recommended reading

Papers of particular interest, published within the period of review, have been highlighted as:

• of special interest
•• of outstanding interest
